# Retrospective Analysis of Vaccination Status and Predominant Viral Variants in Patients Hospitalized with COVID-19 in Reus, Spain

**DOI:** 10.3390/v15040886

**Published:** 2023-03-30

**Authors:** Simona Iftimie, Ana F. López-Azcona, María José Lozano-Olmo, Àngels Naval-Ferrando, Vicent Domingo-Cortés, Helena Castañé, Andrea Jiménez-Franco, Anna Hernández-Aguilera, Carmen Guilarte, Francesc Riu, Jordi Camps, Jorge Joven, Antoni Castro

**Affiliations:** 1 Department of Internal Medicine, Hospital Universitari de Sant Joan, Institut d’Investigació Sanitària Pere Virgili, Department of Medicine and Surgery, Universitat Rovira i Virgili, Av. Dr. Josep Laporte 2, 43204 Reus, Spain; 2 Unitat de Recerca Biomèdica, Hospital Universitari de Sant Joan, Institut d’Investigació Sanitària Pere Virgili, Department of Medicine and Surgery, Universitat Rovira i Virgili, Av. Dr. Josep Laporte 2, 43204 Reus, Spain; 3 Department of Pathology, Hospital Universitari de Sant Joan, Institut d’Investigació Sanitària Pere Virgili, Av. Dr. Josep Laporte 2, 43204 Reus, Spain

**Keywords:** breakthrough infection, COVID-19, pandemic, SARS-CoV-2, vaccines, vaccination

## Abstract

SARS-CoV-2 infection in already-vaccinated individuals is still possible and may require hospitalization. The aim of the present study was to evaluate the clinical evolution of patients with COVID-19 admitted to a public hospital. The outcomes were assessed in relation to the predominant viral variant and the vaccination status. This retrospective study was performed on 1295 COVID-19-positive patients who attended a 352-bed university hospital between 2021 and 2022. Clinical variables and vaccination status were recorded. Of the patients, 799 had not been vaccinated (NV, 61.7%), 449 were partially vaccinated (PV, 34.7%), and 47 were completely vaccinated (CV, 3.6%). The mean age of the CV patients was significantly higher than that of PV and NV. Additionally, they had higher percentages of chronic diseases. The outcomes depended on age but not on vaccination status. There were 209 patients admitted during the Omicron-infection period, of whom 70 (33.5%) were NV, 135 (64.6%) were PV, and 4 (1.9%) were CV. In conclusion, correct vaccination greatly reduces the risk of acquiring severe COVID-19. Partial vaccination does not guarantee protection of the population. This highlights the need for continuous vaccination promotion with all recommended doses, while also investigating alternative treatments for those patients who do not respond to the vaccines.

## 1. Introduction

With the appearance of COVID-19, humanity has been able to identify a global pandemic from its very inception and to evaluate the changes in its geographical distribution in real time, i.e., the epidemiology and biological evolution of the infectious organism. Further, the development of effective vaccines within a few months was an unprecedented scientific and technological achievement [1]. Currently, the pandemic has characteristic features that differentiate it from the original infection. On the one hand, new and more transmissible variants have appeared, while the success of vaccination campaigns has varied in many countries, and as such, the outcomes are hard to predict a priori [2,3]. Understanding the impact of the strategies on the evolution of the disease is essential if definitive treatments are to be defined on a global scale, while alternative treatments need to be found for patients who do not respond to vaccination [4]. A total of 5 SARS-CoV-2 variants of concern have been identified to date: alpha (B.1.1.7 and descendant lineages), beta (B.1.351), gamma (P.1), delta (B.1.617.2 and AY lineages), and omicron (B.1.1.529 and BA lineages). The delta variant was first identified in the spring of 2021 and rapidly replaced the other variants to achieve global dominance by the summer of 2021 [5]. Several studies in the United Kingdom reported an increased risk of hospitalization for those infected with the delta variant compared with the alpha [6,7,8]. The omicron variant was identified in November 2021 and became dominant by the end of December 2021 [9]. The overall risk of hospital admission among those infected with the omicron variant appears to be lower than among those infected with the delta variant [10]. However, studies have suggested reduced vaccine effectiveness against infection and hospital admissions for omicron compared with earlier variants. For example, a case-control study in 21 hospitals across the United States found that three vaccine doses were required to achieve protection against omicron, similar to the protection that two doses provided against the delta and alpha variants [2].

The campaign for vaccination against COVID-19 began in Spain by the end of December 2020. By October 2022, >95,000,000 doses had been administered to a population of approximately 47,000,000 people, and 92.8% of the population > 12 years of age had completed the vaccination schedule. In autumn 2021, a third booster dose was administered to 26,000,000 people, and the campaign for the administration of a fourth dose is currently being launched [11]. These data make Spain, as with the other countries of the European Union, among those with the highest vaccination rates in the world. All the data collected to date agree that vaccination prevents the development of severe COVID-19 in adults and is associated with a decrease in hospitalizations. Boosters of bivalent vaccines have been shown to be effective in preventing medically attended COVID-19 compared with no vaccination and providing additional protection compared with past monovalent vaccination in a nine-state American study by the VISION Network [12]. Moreover, unvaccinated adults were more likely to be hospitalized compared with vaccinated adults, while hospitalization rates were lowest in those who had received a booster dose in another study by the COVID-19-Associated Hospitalization Surveillance Network [13]. However, SARS-CoV-2 infection in those vaccinated is still possible, and although most remain asymptomatic or mild, serious infections requiring hospitalization can also occur [14].

The aim of this study was to evaluate, in a Spanish public hospital, the clinical evolution of patients with COVID-19 infection in relation to the predominant viral variant and their vaccination status. Here, we show the real experience of a university center throughout the COVID-19 pandemic and in the context of compliance with anti-SARS-CoV-2 vaccination recommendations in a country with high vaccination rates.

## 2. Materials and Methods

This retrospective study was conducted on all COVID-19-positive patients who had attended the Hospital Universitari de Sant Joan de Reus between 17 January 2021 and 16 March 2022. This is a reference hospital with 352 beds located in a medium-sized city (100,000 inhabitants) that offers health coverage to the surrounding counties that are mainly rural, with a total population of about 250,000. Included in the catchment area are several primary care centers and nursing homes. According to protocol, all patients who came to the hospital for admission, regardless of the reason for admission or whether they had viral symptoms or were treated in the Emergency Department, underwent a reverse transcriptase polymerase chain reaction (RT-PCR) for SARS-CoV-2. The aim was to isolate positive cases to pre-empt nosocomial infections in other patients. Therefore, the only inclusion criteria were to be at least 18 years old and to have attended the hospital where an RT-PCR was performed with a positive result, regardless of whether the patient was admitted to the ward or was visited in the Emergency Department and referred home after treatment. RT-PCR tests were carried out using the Procleix^®^ method in a Panther automated extractor and amplifier (Grifols Laboratories, Barcelona, Spain) or the VIASURE SARS-CoV-2 Real-Time PCR Detection Kit (CerTest Biotec, Zaragoza, Spain). All experimental protocols were approved by the Comitè d’Ètica i Investigació en Medicaments (Institutional Review Board) of the institution (Resolution CEIM 040/2018, amended on 16 April 2020), and methods were carried out in accordance with all relevant guidelines and regulations. The clinical and demographic data of the patients were collected from the computerized medical records by the research staff. Team members reviewed the records one by one. The McCabe score was calculated as an index of clinical prognosis [15] and the Charlson index as a way of categorizing the patients’ comorbidities [16]. Twenty-nine duplicate cases (more than one PCR performed, or more than once with COVID-19 infection) were not included in the study.

For the purposes of this article, the patients were segregated into three groups: Completely vaccinated (CV) patients were those who had received two doses of Comirnaty^®^ (Pfizer/BioNTech), AZD1222^®^ (AstraZeneca), or Spikewax^®^ Moderna vaccines or combinations of them, or one dose of the Jcovden^®^ (Janssen) vaccine and had a positive PCR test between two weeks and four months of the last dose, the period in which the immune response has been reported to be adequate [17]. Partially vaccinated (PV) patients were those who had received one dose of any of the vaccines recommended or patients who had received a complete course of vaccines but had a positive PCR within two weeks or four months after the last dose. Not vaccinated (NV) were those patients who had not received any vaccine dose. For comparisons, we separated the patients based on the predominant strain in each period and defined three “time windows”: the alpha period, comprising 17 January and 11 July 2021; the delta period, comprising 12 July 2021 and 2 January 2022; and the omicron period, comprising 2 January 2022 and 16 March 2022. Defining these windows was based on official data from the Spanish Ministry of Health [18]. Except where otherwise indicated, results are given as numbers and percentages, or as means and standard deviations. The χ2 test was used to test for differences in categorical data. This test is used to compare the proportions of different groups or to test for independence between two categorical variables. Student’s *t*-test was used to compare the means between the two groups. This test is appropriate when the variables are continuous and normally distributed. Logistic regression was used to model the relationship between a binary outcome variable and one or more predictor variables. It is appropriate when the outcome variable is binary or dichotomous and the predictor variables are continuous or categorical. The statistical significance was set at *p*  <  0.05. All calculations were made using the SPSS 25.0 statistical package (SPSS Inc., Chicago, IL, USA).

## 3. Results

A total of 1295 patients were treated during the study period. Of these, 799 were NV (61.7%), 449 were PV (34.7%), and 47 were CV (3.6%). Only one patient who had received the third dose acquired COVID-19 within the period of peak immune response and had to be hospitalized. The clinical characteristics of the patients are summarized in Table 1. The mean age of the CV patients was significantly higher than that of those who had not received the complete regimen and this, in turn, was higher than that of the unvaccinated patients. Moreover, they had higher percentages of comorbidities associated with age, such as type 2 diabetes mellitus, cardiovascular disease, chronic lung, kidney, neurological diseases, or cancer. In agreement with these data, the values of the Charlson and McCabe indices were higher in hospitalized vaccinated patients. The percentage of patients admitted to the Intensive Care Unit (ICU) was lower, but that of those admitted to the Geriatric Medicine Department (GMD) was higher. In keeping with the more advanced age and more comorbidities of those vaccinated, the percentage of deaths was correspondingly higher.

**Figure 1:**
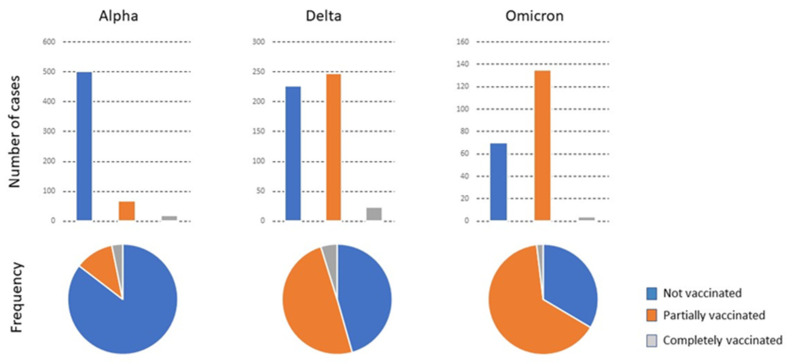
The total number of patients admitted with the predominant variant, their vaccination status, and the frequency of each status relative to the total number of infected individuals.

**Figure 2:**
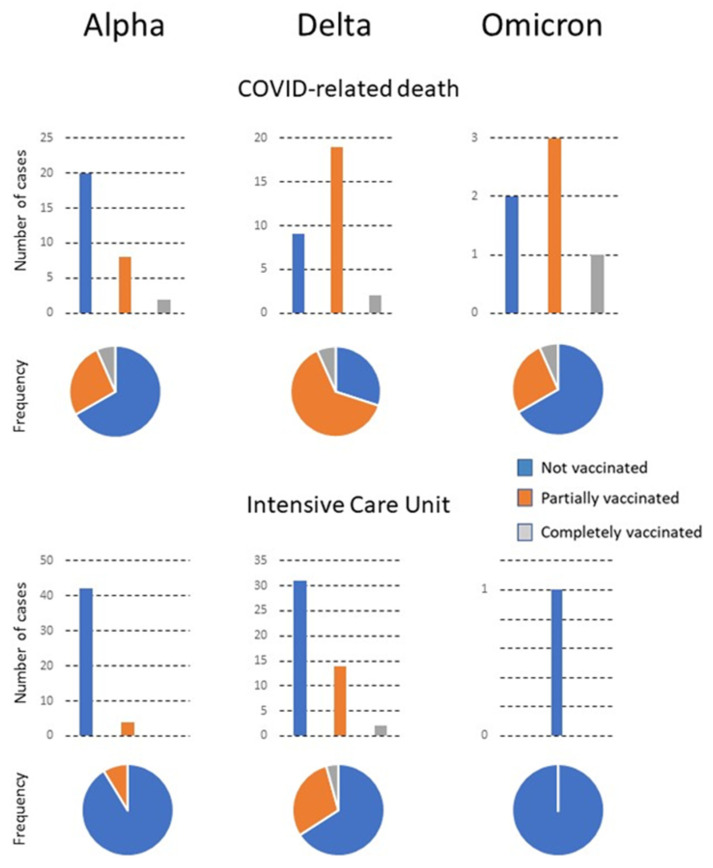
The number of patients with COVID-related death or requiring admission to the Intensive Care Unit (ICU). Numbers are shown in relation to the predominant variant, the vaccination status, and the frequency of each status with respect to the total number of infected individuals.

The variations in the characteristics of patients in relation to the predominant virus variant and the vaccination status are summarized in Figure 1 and Figure 2 and shown in detail in Table S1. In the alpha period, when the vaccination campaign was just beginning, the percentage of PV and CV patients was initially low. The mean age of CV patients admitted was higher than that of PV and NV patients. The percentage of individuals with a smoking habit was higher. CV patients had fewer symptoms but more comorbidities, especially cardiovascular disease, type 2 diabetes mellitus, chronic neuromuscular disease, and cancer. The GMD received the most CV patients for treatment, while NV and PV patients were mostly treated in Internal Medicine. The percentage of deaths of CV patients was higher, although the deaths occurred mostly in the ward, not in the ICU. During the delta period, the vaccination campaign had already advanced considerably, and the number of PV and CV patients correspondingly increased. The mean age of the CV or PV patients was higher than that of the NV. Symptoms were similar in the three subgroups of patients, but comorbidities were higher in CV patients, especially those with cardiovascular disease, chronic kidney or liver disease, and cancer.

The most frequent admissions in the three subgroups were into Internal Medicine. Few patients were admitted to GMD, which was probably linked to the lower mean age of CV patients in the delta compared to the alpha period. The omicron period was associated with a high vaccination rate. Of note is that the number of CV patients admitted during this period was very low, while that of PV was considerably higher. Few reliable statistical analyses can be made with the results of the CV due to the low numbers, except that the age was significantly higher than that of the other patients. Additionally, two were admitted to the Emergency Department and a further two to GMD. There were no admissions to the ICU or Internal Medicine Department. The PV patients were older than the NV patients, had a higher percentage of smokers, and had more comorbidities of cardiovascular disease, type 2 diabetes mellitus, chronic lung, kidney, or neuromuscular diseases, and concomitant infections. These patients were treated mostly in the Emergency Department, followed in numbers by the Outpatient Clinics and Internal Medicine. Mortality tended to decrease with time and the appearance of new viral variants circulating in the population. No significant differences in outcomes were observed in relation to the brand of vaccine administered, except for a greater number of PV patients given the Janssen vaccine who had to be admitted to the ICU. However, the numbers are too low for a reliable statistical analysis (Table 2).

Table 3 shows a logistic regression analysis to identify the factors that showed an independent and statistically significant association with the main outcomes. The determinants of death were age, respiratory failure, and acute kidney failure. The determinants of admission to the ICU were age, respiratory failure, acute respiratory distress syndrome, and concomitant infection. Vaccination status was not significantly associated with these outcomes.

## 4. Discussion

The results of the study are consistent with the well-established concept that complete vaccination is highly effective in protecting against severe COVID-19 Indeed, only 3.6% of the hospitalized patients had received the complete regimen and had acquired the disease during the period considered to be of the maximum immune response. Additionally, only one patient had received the third booster dose. COVID-19 infection, despite full vaccination, is termed a breakthrough infection and, although possible, is considered rare [19,20]. By July 2021, >40,000 SARS-CoV-2 breakthrough infections had been reported in the USA, and 8% of hospitalizations were of fully vaccinated patients [19]. One of the beneficial effects of vaccines described is their ability to reduce the viral load in breakthrough infections and to favor a faster viral load drop [21,22]. Full vaccination status may also decrease transmission as it provides a shorter period of contagion. Transmission studies have suggested that vaccinated individuals are less likely to transmit SARS-CoV-2. This reflects the delta variant, while data on omicron breakthrough rates are as yet sparse [22].

In the current study, the mean age of the CV patients was considerably higher than that of the NV patients and, consequently, so were the number of age-associated comorbidities, especially cardiovascular disease and cancer. These results are similar to those reported by other authors in Spain and other countries [13,23,24,25]. A comprehensive study carried out in the USA showed that monthly hospitalization rates were 17.7 times higher in unvaccinated persons compared with vaccinated persons in May 2021, and that vaccinated hospitalized patients with COVID-19 were older than those who were not vaccinated and, also, had more underlying medical conditions [13]. These data are consistent with our logistic regression analysis indicating that age, cardiovascular disease, and some clinical symptoms, but not vaccination status, are factors independently associated with patient death or admission to the ICU. Indeed, the current study showed that the percentage of mortality was higher in the vaccinated group than in the non-vaccinated group, while the percentage admitted to the ICU was similar. These results may seem counter-intuitive, but other investigators have reached similar conclusions [23,26,27]. The immune response of the elderly is considerably lower than that of young adults. Individuals most at risk of severe disease are those having underlying medical conditions, those who are immunocompromised, and those residing in long-term care facilities, i.e., precisely those who are least likely to mount an adequate immune response to the vaccine [28,29]. These results indicate the clinical need to perform post-vaccination serology in the elderly and immunosuppressed to monitor the immune response to the vaccine.

No clear relationship was found between the commercial brand of the administered vaccine and death or admission to the ICU, i.e., no clear demonstration could be made that one vaccine is better than the others. The only statistically significant difference was a higher percentage of patients who had received the Janssen vaccine requiring admittance to intensive care. In addition, one of the two patients of Chinese origin who had been vaccinated in their own country with the Sinovac vaccine also required intensive care. However, the numbers are too small to draw reliable conclusions.

A novelty of this study has been to present the clinical data and vaccination status of the patients based on the predominant variant of SARS-CoV-2 in each population infection period. Some features have remained constant despite changes to the prevailing variant. During all periods, CV or PV patients admitted to the hospital were older than NV patients, had a higher percentage of smokers, and had more age-related chronic diseases. The mean hospital stays were similar, and the main symptoms were consistently pneumonia, fever, dyspnea, and respiratory failure. However, a major difference is that in the omicron period, the number of CV patients admitted was very low, and their disease was much less severe in terms of symptoms, treatments, ICU admission, and death. This finding supports the concept that COVID-19 vaccines effectively prevent hospitalizations in all adults and protect patients from developing the serious disease [13]. COVID-19 vaccination is an essential tool for preventing morbidity and mortality from COVID-19. A high number of hospitalized cases among vaccinated persons occurred in patients with medical fragility who were older and had comorbidities that possibly affected their immune systems. Indeed, vaccine effectiveness is lower in immunocompromised persons [28,29,30] and repeated booster vaccinations have been vital to avoid COVID-19 hospitalizations [3].

This study has several limitations. This is a single-centered study in a medium size hospital that covers a relatively small geographical area and, as such, the generalizability of the results cannot be advocated. This study was performed in patients with a COVID-19-positive RT-PCR, independently of the reason for admission to the hospital. As such, they had been hospitalized with COVID-19, and not necessarily because of COVID-19. Surgical or trauma patients were not included because non-urgent operations in COVID-19-positive patients were postponed until their RT-PCR scores were negative. In addition, the retrospective nature of the study based on the review of clinical databases is not conducive to highly specific research outcomes since, inevitably, some data may not have been retrieved that could have the potential to impact the outcomes observed. In particular, the viral variant of each patient had not been sequenced, so the assumption is that the patients were infected with the predominant variant current at that time. The antibody titer had not been analyzed either, so the individual degree of immune response can only be assumed. Therefore, the results must be viewed with caution. However, the results obtained are relevant since they could be representative of many similar centers and regions in Western Europe and the Mediterranean area, yet little information has been available on this issue.

## 5. Conclusions

The data show that in our study population, recommended vaccination was associated with a much lower rate of hospitalized patients. This seems to apply to all variants of SARS-CoV-2 that have appeared to date and are even more so for the current omicron variant. However, partial or incomplete vaccination is not effective in protecting the population. The correct application of COVID-19 vaccines prevents hospitalizations in adults, and vaccination is an essential tool for hindering morbidity and mortality from COVID-19. However, our results indicate that there is still a small percentage of the population in which vaccines are not sufficiently effective. These individuals were usually older, had several age-associated chronic diseases, and their outcomes depended on their age and not on their vaccination status. These results suggest that clinicians, public health experts, and health policymakers should continue to promote vaccination with all recommended doses for eligible individuals in the population. Further research on alternative treatments for those patients who do not respond to the current vaccines is still needed.

## 6. Perspectives

It is our belief that forthcoming investigations will depend on the progression of the pandemic. As of now, it appears to be well-contained and on the decline, particularly in Western countries. Nevertheless, the possibility of novel viral variants evading current vaccines cannot be discounted. If such a scenario were to arise, resulting in additional waves of severe infections and heightened fatality rates, it would be valuable to conduct prospective studies across multiple medical centers. To achieve this objective or prepare for new outbreaks of infectious origin, nations should establish necessary mechanisms for inter-hospital coordination, with the imperative of sharing medical records and databases. Furthermore, research on new treatments must persist for individuals of advanced age or with underlying health conditions who are unable to mount an adequate immune response or respond appropriately to existing vaccines.

## Figures and Tables

**Table 1:** Demographic and clinical characteristics of the patients studied.

	Not Vaccinated (*n* = 799)	Partially Vaccinated (*n* = 449)	Completely Vaccinated (*n* = 47)
**Demography**			
Age	49.1 (25.1)	62.9 (20.3) ^c^	69.4 (17.2) ^c,d^
Sex, female	441 (55.2)	238 (53.0)	28 (59.6)
Smoking habit	118 (14.8)	95 (21.1) ^b^	18 (38.3) ^c,e^
Alcohol intake	46 (5.8)	36 (8.0)	9 (19.1) ^b,d^
Symptoms			
Pneumonia	413 (51.7)	180 (40.1) ^c^	15 (31.9) ^b^
Rhinorrhea	161 (20.2)	80 (17.8)	7 (14.9)
Cough	337 (42.2)	167 (37.2) ^a^	11 (23.4) ^b,d^
Fever	461 (57.7)	212 (47.2) ^c^	17 (36.2) ^b^
Chills	27 (3.4)	26 (5.8) ^a^	1 (2.1)
Dyspnea	418 (52.3)	224 (49.9)	18 (38.3) ^a^
General discomfort	355 (44.4)	200 (44.5)	19 (40.4)
Vomiting	67 (8.4)	22 (4.9) ^a^	3 (6.4)
Diarrhea	96 (12.0)	37 (8.2) ^a^	5 (10.6)
Anosmia	57 (7.1)	17 (3.8) ^a^	2 (4.3)
Ageusia or dysgeusia	57 (7.1)	10 (2.2) ^c^	2 (4.3)
Odynophagia	72 (9.0)	48 (10.7)	1 (2.1) ^d^
Headache	153 (19.1)	61 (13.6) ^b^	3 (6.4) ^a^
Anorexia or hyporexia	48 (6.0)	17 (3.8)	3 (6.4)
Myalgia	124 (15.5)	54 (12.0)	3 (6.4)
Arthralgia	80 (10.0)	32 (7.1)	2 (4.3)
Respiratory insufficiency	327 (40.9)	133 (29.6) ^c^	14 (29.8)
Pulmonary embolism	26 (3.3)	6 (1.4) ^a^	0
ARDS	41 (5.1)	11 (2.4) ^a^	1 (2.1)
Dysuria	1 (0.1)	2 (0.4)	0
Acute kidney failure	37 (4.6)	27 (6.0)	3 (6.4)
Other symptoms	183 (22.9)	79 (17.6) ^a^	3 (6.4) ^b,d^
Ageusia or dysgeusia	57 (7.1)	10 (2.2) ^c^	2 (4.3)
Odynophagia	72 (9.0)	48 (10.7)	1 (2.1) ^d^
**Comorbidities**			
Type 2 diabetes mellitus	122 (15.3)	105 (23.4) ^c^	13 (27.7) ^a^
Cardiovascular disease	326 (40.8)	262 (58.4) ^c^	36 (76.6) ^c,d^
Chronic liver disease	37 (4.6)	28 (6.2)	7 (14.9) ^b,d^
Chronic lung disease	97 (12.1)	105 (23.4) ^c^	9 (19.1)
Chronic kidney disease	55 (6.9)	64 (14.3) ^c^	11 (23.4) ^b^
CNMD	51 (6.4)	80 (17.8) ^c^	8 (17.0) ^a^
Immunosuppressed	16 (2.0)	9 (2.0) ^c^	0
Cancer	72 (9.0)	72 (16.0) ^c^	17 (36.2) ^c,f^
Obesity	147 (18.4)	75 (16.7)	8 (17.0)
Pregnancy	9 (1.1)	1 (0.2)	0
Postpartum	5 (0.6)	2 (0.4)	0
Concomitant infection	109 (13.6)	64 (14.2)	5 (10.6)
Charlson index 0 1 2 3 4 5 6 7 9	533 (66.7) 105 (13.1) 79 (9.9) 45 (5.6) 22 (2.8) 10 (1.2) 3 (0.4) 2 (0.3) 0	200 (44.6) ^c^ 74 (16.6) 64 (14.2) 49 (10.9) 37 (8.2) 12 (2.7) 7 (1.6) 5 (1.1) 1 (0.1)	12 (25.5) ^c^ 6 (12.8) 10 (21.3) 6 (12.8) 11 (23.4) 1 (2.1) 1 (2.1) 0 0
McCabe score NFD UFD RFD	727 (92.1) * 61 (7.8) 1 (0.1)	341 (75.9) ^c^ 106 (23.6) 2 (0.5)	33 (70.2) ^c^ 14 (29.8) 0
COVID-19-related death	31 (3.9)	30 (6.7) ^a^	5 (10.6) ^a^
Treatments			
NIMV	14 (1.8)	8 (1.8)	1 (2.1)
IMV	59 (7.4)	17 (3.8) ^b^	1 (2.1) ^c,e^
HFOT	87 (10.9)	20 (4.4)^c^	1 (2.1)
COT	399 (49.9)	207 (46.1)	21 (44.7) ^b,e^
Anticoagulants	423 (52.9)	236 (52.6)	28 (59.6)
Corticoids	438 (54.8)	219 (48.8) ^a^	26 (55.3)
Remdesivir	43 (5.4)	17 (3.8)	3 (6.4)
Days of hospitalization	8.5 (15.4)	10.1 (18.7)	9.8 (10.1)
Hospitalization site Internal Medicine Surgery Emergency Geriatrics Oncology Outpatient clinics ICU Pediatric emergencies Traumatology Gynecology Urology Gastroenterology	326 (40.8) 4 (0.5) 197 (24.6) 41 (5.1) 9 (1.1) 66 (8.3) 73 (9.1) 74 (9.3) 3 (0.4) 6 (0.8) 0 0	161 (35.9) ^c^ 5 (1.1) 131 (29.2) 60 (13.4) 10 (2.2) 49 (10.9) 19 (4.2) 2 (0.4) 6 (1.3) 3 (0.7) 2 (0.4) 1 (0.2)	16 (34.0) ^c^ 1 (2.1) 9 (19.1) 11 (23.4) 4 (8.5) 3 (6.4) 2 (4.3) 0 0 1 (2.1) 0 0

Data are shown as the number of cases and percentages or as means and SD. Statistical significance was calculated using the χ2 test (qualitative data) or Student’s *t*-test (quantitative data). ARDS—acute respiratory distress syndrome; CNMD—chronic neurological or neuromuscular disease; COT—conventional oxygen therapy; HFOT—high-flow oxygen therapy; ICU—Intensive Care Unit; IMV—invasive mechanical ventilation; NFD—non-fatal disease; NIMV—non-invasive mechanical ventilation; RFD—rapidly fatal disease; UFD—ultimately fatal disease. * *n* = 789. ^a^ *p* < 0.05, ^b^ *p* < 0.01, ^c^ *p* < 0.001, with respect to non-vaccinated patients; ^d^ *p* < 0.05, ^e^ *p* < 0.01, ^f^ *p* < 0.001, with respect to patients with incomplete vaccination.

**Table 2:** Patients with COVID-related death or admitted to the Intensive Care Unit were segregated with respect to the brand of vaccine administered.

	Partially Vaccinated			Completely Vaccinated		
Brand	Total	Deceased	Intensive Care	Total	Deceased	Intensive Care
Pfizer	325	24 (7.4)	10 (3.1)	35	4 (11.4)	1 (2.8)
AstraZeneca	59	5 (8.5)	3 (5.1)	8	0 (0)	0 (0)
Moderna	38	1 (2.6)	1 (2.6)	4	1 (0.25)	1 (0.25)
Janssen	25	0 (0)	4 (16.0) ^a^	0	0 (0)	0 (0)
Sinovac	2	0 (0)	1 (50.0)	0	0 (0)	0 (0)

The results are presented as numbers and percentages of the totals (in parenthesis).^a^ *p* < 0.05 with respect to the Pfizer vaccine.

**Table 3:** Logistic regression analysis of the variables associated with COVID-19-related death, or admission into the Intensive Care Unit.

	Death ^1^				Intensive Care Unit ^2^			
Variable	B	SE	Exp (B)	*p*-Value	B	SE	Exp (B)	*p*-Value
Age	0.048	0.015	1.049	0.002	−0.040	0.012	0.961	0.001
Sex	−0.390	0.344	0.677	0.256	0.437	0.330	1.547	0.186
Smoking habit	0.477	0.399	1.611	0.231	−0.216	0.365	0.806	0.553
Alcohol intake	0.009	0.549	1.010	0.986	0.450	0.534	1.569	0.399
Pneumonia	0.719	0.514	2.052	0.162	0.443	0.727	1.557	0.543
Rhinorrhea	−0.090	0.534	0.914	0.866	−1.340	0.519	0.262	0.010
Cough	−0.037	0.347	0.964	0.916	0.356	0.324	1.428	0.271
Fever	−0.090	0.332	0.914	0.787	−0.261	0.347	0.770	0.452
Chills	−0.584	0.973	0.558	0.548	−0.758	0.954	0.469	0.427
Dyspnea	0.250	0.602	1.284	0.678	1.136	0.762	3.113	0.136
General discomfort	−0.450	0.334	0.638	0.179	−0.087	0.327	0.916	0.789
Vomiting	−0.862	0.895	0.422	0.336	−0.466	0.579	0.627	0.421
Diarrhea	−0.263	0.572	0.768	0.645	−0.353	0.453	0.703	0.436
Anosmia	−13.215	3271.063	0.000	0.997	−1.725	1.653	0.178	0.297
Ageusia or dysgeusia	−10.886	3315.194	0.000	0.997	1.187	1.647	3.277	0.471
Odynophagia	0.599	0.773	1.821	0.438	−0.202	0.574	0.817	0.725
Headache	−0.667	0.706	0.513	0.345	0.854	0.367	2.350	0.020
Anorexia or hyporexia	−0.465	0.855	0.628	0.587	0.642	0.633	1.900	0.311
Myalgia	−0.750	0.987	0.472	0.447	0.065	0.626	1.068	0.917
Arthralgia	1.017	1.068	2.766	0.313	0.216	0.667	1.241	0.746
Respiratory insufficiency	1.779	0.500	5.926	<0.001	2.762	0.759	15.825	<0.001
Pulmonary embolism	0.559	0.757	1.748	0.461	0.924	0.605	2.520	0.127
ARDS	0.845	0.581	2.328	0.146	1.982	0.431	7.259	<0.001
Dysuria	−18.287	20,958.238	0.000	0.999	−20.502	19,068.955	0.000	0.999
Acute kidney failure	0.943	0.423	2.567	0.026	1.132	0.473	3.103	0.017
Other symptoms	−0.056	0.380	0.946	0.883	−0.031	0.357	0.969	0.930
T2DM	0.570	0.325	1.769	0.079	0.108	0.375	1.114	0.773
Cardiovascular disease	1.878	0.776	6.543	0.016	0.608	0.400	1.838	0.128
Chronic liver disease	0.363	0.496	1.438	0.464	−0.148	0.509	0.863	0.772
Chronic lung disease	0.168	0.354	1.183	0.636	0.165	0.383	1.180	0.666
Chronic kidney disease	0.220	0.369	1.246	0.551	0.378	0483	1.459	0.435
CNMD	0.446	0.398	1.562	0.262	−1.263	0.798	0.283	0.114
Immunosuppressed	0.099	0.842	1.104	0.906	−0.059	1.051	0.943	0.955
Cancer	0.485	0.367	1.624	0.187	0.546	0.444	1.727	0.219
Obesity	−0.083	0.387	0.920	0.830	0.484	0.327	1.623	0.139
Pregnancy	−12.431	11,017.879	0.000	0.999	−14.654	11,667.424	0.000	0.999
Postpartum	−11.810	12,520.776	0.000	0.999	−14.881	13,882.766	0.000	0.999
Concomitant infection	0.632	0.342	1.881	0.065	1.860	0.339	6.424	<0.001
Vaccinated before COVID-19	0.277	0.277	1.320	0.317	−0.277	0.348	0.758	0.427
Constant	−10.258	1.457	0.000	<0.001	−5.163	0.880	0.000	<0.001

^1^ Model summary: log-likelihood (−2) = 313.923; *r^2^* Cox and Snell = 0.148; *r^2^* Nagelkerke = 0.447; *p* < 0.001. ^2^ Model summary: log-likelihood (−2) = 342.942; *r^2^* Cox and Snell = 0.226; *r^2^* Nagelkerke = 0.556; *p* <0.001. ARDS—acute respiratory distress syndrome; B—non-standardized β coefficient; CNMD—chronic neurological or neuromuscular disease; SE—standard error of B; T2DM—type 2 diabetes mellitus.

## Data Availability

The datasets used and/or analyzed during the current study are available upon reasonable request to the corresponding authors.

## Supplementary Materials

The following supporting information can be downloaded at: https://www.mdpi.com/article/10.3390/v15040886/s1, Table S1: Demographic and clinical characteristics of the patients segregated with respect to the predominant viral variant.
