# Supplementary material for: Retrospective Analysis of Vaccination Status and Predominant Viral Variants in Patients Hospitalized with COVID-19 in Reus, Spain
Source: Viruses. 2023 Mar 30;15(4):886. doi: 10.3390/v15040886 (PMC10143314; doi:10.3390/v15040886)
Supplement: Supplementary file 1 [file viruses-15-00886-s001.zip › Supplementary Table S1-vir.pdf]

**Table S1.** Demographic and clinical characteristics of the patients segregated with respect to the predominant viral variant

| Time window | Alpha | | | Delta | | | Omicron | | |
|---|---|---|---|---|---|---|---|---|---|
| **Vaccination status** | **Not vaccinated *n* = 502** | **Partially vaccinated *n*= 67** | **Completely vaccinated *n* = 19** | **Not vaccinated *n* = 227** | **Partially vaccinated *n* = 247** | **Completely vaccinated *n* = 24** | **Not vaccinated *n* = 70** | **Partially vaccinated *n* = 135** | **Completely vaccinated *n* = 4** |
| **Demography** | | | | | | | | | |
| Age | 53.1 (23.1) | 63.7 (17.8)[c] | 77.4 (14.1)[c] | 42.9 (25.5)[i] | 65.7 (20.2)[c] | 63.9 (15.8)[c,h] | 34.7 (27.9)[I,j] | 57.3 (20.8)[c,g,l] | 64.0 (27.0)[a] |
| Sex, female | 275 (54.8) | 34 (50.7) | 11 (57.9) | 129 (56.8) | 132 (53.4) | 17 (70.8) | 37 (52.9) | 72 (53.3) | 0[j] |
| Smoking habit | 87 (17.3) | 16 (23.9) | 7 (36.8)[a] | 29 (12.8) | 48 (19.4)[a] | 11 (45.8)[c,e] | 2 (2.9)[h,j] | 31 (23.0)[c,h,j] | 0 |
| Alcohol intake | 34 (6.8) | 5 (7.5) | 2 (10.5) | 11 (4.8) | 17 (6.9) | 7 (29.2)[c,f] | 1 (1.4) | 14 (10.4)[a] | 0 |
| **Symptoms** | | | | | | | | | |
| Pneumonia | 260 (51.8) | 38 (56.7) | 3 (15.8)[b,e] | 135 (59.5)[g] | 112 (45.3)[b,g] | 12 (50.0)[g] | 18 (25.7)[i,l] | 30 (22.2)[i,k] | 0 |
| Rhinorrhea | 71 (14.1) | 15 (22.4) | 1 (5.3) | 59 (26.0)[i] | 28 (11.3)[c,g] | 5 (20.8) | 31 (44.3)[i,k] | 37 (27.4)[a,l] | 1 (0.25) |
| Cough | 180 (35.9) | 21 (31.3) | 2 (10.5) | 122 (53.7)[i] | 84 (34.0)[c,i] | 7 (29.2)[a] | 35 (50.0)[j] | 62 (45.9)[g] | 2 (0.50) |
| Fever | 279 (55.6) | 38 (56.7) | 6 (31.6)[d] | 147 (64.8)[g] | 127 (51.4)[b,g] | 10 (41.7)[a] | 35 (50.0) | 47 (34.8)[a,h,j] | 1 (0.25) |
| Chills | 11 (2.2) | 3 (4.5) | 1 (5.3) | 5 (2.2) | 6 (2.4) | 0 | 11 (15.7) | 17 (12.6)[k,l] | 0 |
| Dyspnea | 267 (53.2) | 40 (59.7) | 7 (36.8) | 130 (57.3) | 136 (55.1) | 11 (45.8) | 21 (30.0) | 48 (35.6)[h,l] | 0 |
| General discomfort | 207 (41.2) | 33 (49.3) | 3 (15.8)[a,e] | 112 (49.3)[g] | 101 (40.9)[g] | 13 (54.2)[g] | 36 (51.4) | 66 (48.9) | 3 (0.75)[g] |
| Vomiting | 36 (7.2) | 2 (3.0) | 0 | 24 (10.6) | 14 (5.7) | 3 (12.5) | 7 (10.0) | 6 (4.4) | 0 |
| Diarrhea | 59 (11.8) | 8 (11.9) | 1 (5.3) | 30 (13.2) | 22 (8.9) | 4 (16.7) | 7 (10.0) | 7 (5.2) | 0 |
| Anosomia | 31 (61.8) | 7 (10.4) | 1 (5.3) | 25 (11.0)[g] | 5 (2.0)[c,h] | 1 (4.2) | 1 (1.4) | 5 (3.7) | 0 |
| Ageusia or dysgeusia | 28 (5.6) | 5 (7.5) | 1 (5.3) | 26 (11.5)[h] | 4 (1.6)[c,g] | 1 (4.2) | 3 (4.3) | 1 (0.7)[g] | 0 |
| Odynophagia | 40 (8.0) | 7 (10.4) | 0 | 27 (11.9) | 22 (8.9) | 0 | 5 (7.1) | 19 (14.1) | 1 (0.25) |
| Headache | 96 (19.1) | 14 (20.9) | 1 (5.3) | 48 (21.1) | 29 (11.7)[b] | 1 (4.2) | 9 (12.9) | 18 (13.3) | 1 (0.25) |
| Anorexia or hyporexia | 25 (5.0) | 4 (6.0) | 1 (5.3) | 19 (8.4) | 8 (3.2)[a] | 2 (8.4) | 4 (5.7) | 5 (3.7) | 0 |
| Myalgia | 79 (15.7) | 12 (17.9) | 0 | 39 (17.2) | 30 (12.1) | 1 (4.2) | 6 (8.6) | 12 (8.9) | 2 (0.50)[g,j] |
| Arthralgia | 50 (10.0) | 8 (11.9) | 0 | 24 (10.6) | 11 (4.4) | 1 (4.2) | 6 (8.6) | 3 (2.2)[h,j] | 1 (0.25) |
| Respiratory insufficiency | 203 (40.4) | 27 (40.3) | 4 (21.1) | 113 (49.8)[g] | 86 (34.8)[b] | 10 (41.7)[i] | 11 (15.7) | 20 (14.8)[i,l] | 0 |
| Pulmonary embolism | 18 (3.6) | 1 (1.5) | 0 | 6 (2.6) | 4 (1.6) | 0 | 2 (2.9) | 1 (0.7) | 0 |
| ARDS | 19 (3.8) | 2 (3.0) | 0 | 17 (7.5)[g] | 7 (2.8)[a] | 1 (4.2) | 5 (7.1) | 2 (1.5)[a] | 0 |
| Dysuria | 0 | 0 | 0 | 1 (0.4) | 0 | 0 | 0 | 2 (1.5) | 0 |
| Acute kidney failure | 25 (5.0) | 3 (4.5) | 1 (5.3) | 12 (5.3) | 20 (8.1) | 2 (8.4) | 0 | 4 (3.0)[j] | 0 |
| Other symptoms | 138 (27.5) | 18 (26.9) | 0[e] | 32 (14.1)[i] | 35 (14.2)[g] | 2 (8.4) | 13 (18.6) | 26 (19.3) | 1 (0.25) |
| **Comorbidities** | | | | | | | | | |
| Type 2 diabetes mellitus | 94 (18.7) | 16 (23.9) | 8 (42.2)[a] | 23 (10.1)[h] | 65 (26.3)[c] | 5 (20.8) | 5 (7.1) | 24 (17.8)[a] | 0 |
| Cardiovascular disease | 243 (48.4) | 38 (56.7) | 17 (89.5)[c,e] | 66 (29.1)[h] | 156 (63.2)[c] | 17 (70.8)[c] | 17 (24.3) | 68 (50.4)[c,j] | 2 (0.50) |
| Chronic liver disease | 24 (4.8) | 4 (6.0) | 1 (5.3) | 9 (4.0) | 17 (6.9) | 5 (20.8)[b,d] | 4 (5.7) | 7 (5.2) | 1 (0.25) |
| Chronic lung disease | 64 (12.7) | 13 (19.4) | 3 (15.8) | 31 (13.7) | 63 (25.5)[b] | 6 (25.0) | 2 (2.9) | 19 (14.1)[c] | 0 |
| Chronic kidney disease | 40 (8.0) | 8 (11.9) | 4 (21.1) | 11 (4.8) | 44 (17.8)[c] | 5 (20.8)[a] | 4 (5.7) | 12(8.9)[j] | 2 (0.50) |
| CNMD | 42 (8.4) | 11(16.4)[a] | 6 (31.6)[b] | 9 (4.0)[g] | 5 (2.0)[b,c] | 2 (8.4) | 0 | 16 (11.9)[b,j] | 0 |

| | | | | | | | | | |
|---|---|---|---|---|---|---|---|---|---|
| Immunosuppressed | 13 (2.6) | 1 (1.5) | 0 | 2 (0.8) | 4 (1.6) | 0 | 1 (1.4) | 4 (3.0) | 0 |
| Cancer | 52 (10.4) | 11 (16.4) | 6 (31.6)[b] | 13 (5.7)[g] | 44 (17.8)[c] | 10 (41.7)[c,d] | 7 (10.0) | 17 (12.6) | 1 (0.25) |
| Obesity | 104 (20.7) | 19 (28.4) | 2 (10.5) | 34 (15.0) | 34 (13.8)[h] | 5 (20.8) | 9 (12.9) | 22 (16.3) | 1 (0.25) |
| Pregnancy | 0 | 0 | 0 | 8 (3.5)[i] | 1 (0.4)[a] | 0 | 1 (1.4) | 0 | 0 |
| Postpartum | 0 | 0 | 0 | 4 (1.8)[h] | 0 | 0 | 1 (1.4) | 2 (1.5) | 0 |
| Concomitant infection | 77 (15.3) | 11 (16.4) | 2 (10.5) | 29 (12.8) | 41 (16.6) | 3 (12.5) | 3 (4.3) | 12 (8.9)[j] | 0 |
| Charlson index | | | | | | | | | |
| 0 | 304 (60.6) | 30 (44.8) | 3 (15.8)[c] | 174 (76.7)[h] | 99 (40.1)[c] | 7 (29.2)[c] | 56 (80.0) | 71 (53.4)[a,j,*] | 2 (0.50) |
| 1 | 72 (14.3) | 12 (17.9) | 3 (15.8) | 26 (11.5) | 35 (14.2) | 3 (12.5) | 7 (10.0) | 27 (20.3) | 0 |
| 2 | 60 (12.0) | 11 (16.4) | 4 (21.1) | 15 (6.6) | 35 (14.2) | 5 (20.8) | 4 (5.7) | 18 (13.5) | 1 (0.25) |
| 3 | 39 (7.8) | 5 (7.5) | 2 (10.5) | 6 (2.6) | 37 (15.0) | 4 (16.7) | 0 | 7 (5.3) | 0 |
| 4 | 17 (3.4) | 5 (7.5) | 7 (36.8) | 3 (1.3) | 24 (9.7) | 4 (16.7) | 2 (2.9) | 8 (6.0) | 1 (0.25) |
| 5 | 6 (1.2) | 2 (3.0) | 0 | 2 (0.8) | 8 (3,2) | 1 (4.2) | 0 | 2 (1.5) | 2 (0.50) |
| 6 | 2 (0.4) | 1 (1.5) | 0 | 1 (0.4) | 5 (2.0) | 0 | 1 (1.4) | 0 | 1 (0.25) |
| 7 | 2 (0.4) | 1 (1.5) | 0 | 0 | 4 (1.6) | 0 | 0 | 0 | 0 |
| 9 | 0 | 0 | 0 | 0 | 0 | 0 | 0 | 0 | 0 |
| McCabe score | | | | | | | | | |
| NFD | 454 (90.4) | 51 (76.1)[b] | 12 (63.2)[c] | 215 (94.7) | 174 (70.4)[c] | 17 (70.8)[c] | 68 (97.1) | 116 (85.9)[l] | 4 (1.00) |
| UFD | 48 (9.6) | 16 (23.9) | 7 (36.8) | 11 (4.8) | 73 (29.6) | 7 (29.2) | 2 (2.9) | 17 (12.6) | 0 |
| RFD | 0 | 0 | 0 | 1 (0.4) | 0 | 0 | 0 | 2 (1.5) | 0 |
| COVID-19-related death | 20 (4.0) | 8 (11.9)[a] | 2 (10.5)[b] | 9 (4.0) | 19 (3.4) | 2 (8.4) | 2 (2.9) | 3 (2.2)[g,j] | 1 (0.25) |
| **Treatments** | | | | | | | | | |
| NIMV | 10 (2.0) | 1 (1.5)[a] | 1 (5.3) | 1 (0.4) | 2 (0.8) | 0 | 3 (4.3)[g] | 5 (3.7) | 0 |
| IMV | 34 (6.8) | 6 (9.0) | 0[c] | 23 (10.1) | 8 (3.2)[b] | 1 (4.2) | 2 (2.9) | 3 (2.2) | 0 |
| HFOT | 46 (9.2) | 5 (7.5) | 0 | 36 (15.9)[g] | 11 (4.5)[c] | 1 (4.2) | 5 (7.1) | 4 (3.0) | 0 |
| COT | 256 (51.0) | 38 (56.7) | 8 (42.1)[c] | 130 (57.3) | 132 (53.4) | 12 (50.0) | 13 (18.6)[i,l] | 37 (27.4)[i,l] | 1 (0.25) |
| Anticoagulants | 275 (54.8) | 42 (62.7) | 9 (47.4) | 135 (59.5) | 147 (59.5) | 18 (75%) | 13 (18.6)[i,l] | 47 (34.8)[a,I,l] | 1 (0.25) |
| Corticoids | 282 (56.2) | 45 (67.2) | 11 (57.9) | 137 (59.5) | 131 (53.0) | 14 (58.3) | 19 (27.1)[i,l] | 43 (31.9)[i,l] | 1 (0.25) |
| Remdesivir | 28 (5.6) | 6 (9.0) | 3 (15.8) | 13 (5.7) | 5 (2.0)[a] | 0 | 2 (2.9) | 6 (4.4) | 0 |
| Days of hospitalization | 9.0 (16.1) | 13.3 (23.0)[c] | 10.8 (10.0)[c] | 8.6 (14.5) | 10.9 (18.1)[c] | 8.5 (8.7) | 4.3 (12.4)[g] | 7.0 (17.2)[g,j] | 12.7 (20.0) |
| **Hospitalization site** | | | | | | | | | |
| Internal Medicine | 219 (43.6) | 35 (52.2)[a] | 3 (15.8)[c,e] | 102 (44.9)[c,i] | 103 (41.7)[c] | 13 (54.2)[b,d] | 5 (7.1)[i,l] | 23 (17.0)[c,I,l] | 0[j] |
| Surgery | 3 (0.6) | 3 (4.5) | 0 | 1 (0.4) | 1 (0.4) | 1 (4.2) | 0 | 1 (0.7) | 0 |
| Emergency | 129 (25.7) | 14 (20.9) | 4 (21.1) | 36 (15.9) | 60 (24.3) | 3 (12.5) | 32 (45.7) | 57 (42.2) | 2 (0.50) |
| Geriatrics | 34 (6.8) | 7 (10.4) | 8 (42.1) | 5 (2.2) | 36 (14.6) | 1 (4.2) | 2 (2.9) | 17 (12.6) | 2 (0.50) |
| Oncology | 7 (1.4) | 1 (1.5) | 1 (5.3) | 2 (0.9) | 7 (2.8) | 3 (12.5) | 0 | 2 (1.5) | 0 |
| Outpatient clinics | 48 (9.6) | 2 (3.0) | 3 (15.8) | 11 (4.8) | 22 (8.9) | 0 | 7 (10.0) | 25 (18.5) | 0 |
| ICU | 42 (8.4) | 4 (6.0) | 0 | 31 (13.7) | 14 (5.7) | 2 (8.4) | 0 | 1 (0.7) | 0 |
| Pediatric emergencies | 17 (3.4) | 0 | 0 | 35 (15.4) | 1 (0.4) | 0 | 22 (31.4) | 1 (0.7) | 0 |
| Traumatology | 3 (0.6) | 1 (1.5) | 0 | 0 | 1 (0.4) | 1 (4.2) | 0 | 4 (3.0) | 0 |
| Gynecology | 0 | 0 | 0 | 4 (1.8) | 1 (0.4) | 0 | 2 (2.9) | 2 (1.5) | 0 |
| Urology | 0 | 0 | 0 | 0 | 1 | 0 | 0 | 1 (0.7) | 0 |
| Gastroenterology | 0 | 0 | 0 | 0 | 0 | 0 | 0 | 1 (0.7) | 0 |

Data are presented as number of cases and percentages, or as means and SD. Statistical significance calculated by the $\chi^2$ test (qualitative data) or the Student's *t*-test (quantitative data). ARDS: Acute respiratory distress syndrome; CNMD: Chronic neurological or neuromuscular disease; COT: Conventional oxygen therapy; HFOT: High-flow oxygen therapy; ICU: Intensive Care Unit; IMV: Invasive mechanical ventilation; NFD: Non-fatal disease; NIMV: Non-invasive mechanical ventilation; RFD: Rapidly fatal disease; UFD: Ultimately fatal disease. [a] $p < 0.05$, [b] $p < 0.01$, [c] $p < 0.001$, with respect to non- vaccinated patients; [d] $p < 0.05$, [e] $p < 0.01$, [f] $p < 0.001$, with respect to patients with incomplete vaccination; [g] $p < 0.05$, [h] $p < 0.01$, [i] $p < 0.001$, with respect to patients from the alpha time window; [j] $p < 0.05$, [k] $p < 0.01$, [l] $p < 0.001$, with respect to patients from the delta time window. * $n$ =133.
